# Effective General Practice–Led Interventions to Increase Uptake in Colorectal Cancer Screening: A Scoping Review

**DOI:** 10.1097/PHH.0000000000002367

**Published:** 2026-05-08

**Authors:** Anna M. Kelly, Stephanie C. Walker, Georgia Carney, Kelera Levu, Christopher Horn, Mark A. Jenkins, Natalie Taylor, Eleonora Feletto

**Affiliations:** **Author Affiliations:** The Daffodil Centre, The University of Sydney, and Cancer Council NSW, New South Wales, Australia (Kelly, Walker, Carney, Levu, and Feletto); Walker is now with the School of Clinical Medicine, University of New South Wales, High St, Kensington, New South Wales, Australia; Cancer Elimination Collaboration, School of Public Health, The University of Sydney, New South Wales, Australia (Kelly and Feletto); Cancer Institute NSW, New South Wales, Australia (Horn); Centre for Epidemiology and Biostatistics, Melbourne School of Population and Global Health, University of Melbourne, Victoria, Australia (Jenkins); and Implementation to Impact (i2i), School of Population Health, UNSW, New South Wales, Australia (Taylor).

**Keywords:** colorectal cancer, primary care, general practice, population screening, intervention, implementation

## Abstract

**Context:**

Primary care, specifically general practice, represents the front line of health care. For colorectal cancer (CRC) screening, general practice provides an effective setting to improve participation in organized screening.

**Objective:**

The aim of this study was to review evidence from systematic reviews with meta-analyses (SRMAs) on interventions to increase CRC screening, and from randomized controlled trials (RCTs) on the effectiveness and implementation of general practice–led interventions.

**Design:**

This scoping review included 2 phases: 1) an umbrella review of SRMAs evaluating CRC screening participation after intervention delivery, and 2) a review of RCTs evaluating general practice–led interventions, which were also coded for implementation strategies.

**Eligibility Criteria:**

For the SRMAs, interventions could be designed to increase participation of any type of population-based CRC screening. For the RCTs, only general practice–led interventions designed to increase screening using fecal occult blood tests were included. Studies had to be published in English and available in full text.

**Study Selection:**

We searched MEDLINE, Embase, and PsycInfo, guided by PRISMA guidelines, from May 2016 to August 2023.

**Main Outcome Measure(s):**

All evidence was assessed for intervention effectiveness and, for RCTs, implementation strategies using one implementation science framework and 2 taxonomies.

**Results:**

We identified 15 SRMAs and 12 RCTs, which evaluated individual, primary care/general practice, and population-level interventions. Key interventions including patient reminders, risk assessment tools, and kit outreach improved CRC screening participation. Evidence on multicomponent interventions is promising. None of the included RCTs explicitly outlined implementation strategies.

**Conclusions:**

Our findings highlight the possible effectiveness of individual, primary care/general practice, and population-level interventions and their potential use in combination. However, there was a lack of implementation strategies to support adaptation and sustainability. Future trials should explore how interventions can mobilize general practice to promote CRC screening, incorporating targeted implementation strategies to enhance their real-world application and sustainability.

## Abstract 2

Supplemental Digital Content is Available in the Text.

## INTRODUCTION

Primary care, specifically general practice, is the first point of care for individuals seeking medical attention. Primary care is a promising setting for promoting organized cancer screening programs.^1,2^ Previous studies have demonstrated the effectiveness of general practitioner (GP) endorsement in improving cancer screening participation.^3–6^

Colorectal cancer (CRC) is a global health burden reducible by effective screening.^7,8^ CRC screening can be delivered through stool-based tests, such as fecal occult blood tests (FOBTs)—either an immunochemical fecal occult blood test (iFOBT) or a guaiac fecal occult blood test (gFOBT)—stool DNA tests, or direct visualization tests (eg, colonoscopy).^9^ Organized CRC population screening programs exist worldwide, with reported participation ranging from 16% to 68.2%.^8,10^ They commonly use the noninvasive and cost-effective iFOBT test.^10,11^

General practice involvement could increase participation in organized CRC screening and reduce the CRC burden.^12^ Interventions can be categorized into three groups: 1) population-level interventions, for example, mass media campaigns^13^; 2) primary care–level interventions, for example, GP-targeted education^14,15^; and 3) individual-level interventions, for example, patient-targeted reminders.^16^ Effectiveness of such interventions depends on how well they are implemented and their effect on the offer, uptake, and delivery or self-collection of screening. The implementation of an intervention and the behaviors required to sustain the change are critical to success,^17^ already noted for CRC screening.^18–21^

In the CRC space, little is known about the benefit of general practice–led interventions, defined as interventions led by a GP or primary care practitioner in the general practice setting, across the three intervention groups, or about their implementation strategies.^22^ As global uptake of organized CRC screening remains suboptimal, general practice–led interventions present a valuable opportunity to promote participation. This scoping review synthesized evidence from systematic reviews with meta-analyses (SRMAs) on interventions to increase CRC screening, and from randomized controlled trials (RCTs) on general practice–led interventions to increase CRC screening. The secondary aim was to assess implementation strategies using one implementation science framework and 2 taxonomies.

## METHODS

### Design

The two-phase scoping review assessed SRMAs and RCTs using an iterative approach that allowed for the scope of the search to evolve based on findings from previous phases. Phase 1 was an umbrella review of SRMAs evaluating CRC screening participation after intervention delivery and was not limited to general practice. Phase 2 was a targeted review of RCTs, which evaluated the effectiveness of general practice–led interventions to increase FOBT uptake. The reviews were conducted in line with PRISMA guidelines. The PICOS (Population, Intervention, Comparator, Outcome, Study Design) framework is outlined in Supplementary Tables 1–2, http://links.lww.com/JPHMP/B673. Ethics approval for this study was not required.

### Search strategy

MEDLINE (MEDLINE Epub Ahead of Print, I-Process, and Other Non-Indexed Citations), Embase, and PsycInfo (through OVID) were searched combining text terms and/or database-specific subject headings for “colorectal cancer,” “screening,” and “participation.” Search strategies were developed based on keywords, study type, study design, publication date, publication type, and language restrictions. Searches included all published studies from May 2016 to August 2023 to focus on contemporary evidence. Supplementary Table 3, http://links.lww.com/JPHMP/B673, provides full search strategy.

### Eligibility criteria

We included studies reporting on interventions targeting average-risk adults eligible for population-based CRC screening. For the SRMAs, interventions could be designed to increase participation of any type of primary CRC screening (not follow-up of a positive screening test). For the RCTs, only general practice–led interventions designed to increase FOBT screening as part of an organized program were included. All studies had to be published in English and available in full text. Details are given in Supplementary Tables 4–5, http://links.lww.com/JPHMP/B673.

### Data extraction, analysis, and coding

Three team members were involved in data extraction, analysis, and coding. Inclusion discrepancies were resolved by the Lead Investigator, and initial data extractions were performed independently to assess inter-reliability. Data extracted included study and population characteristics, screening modality, intervention characteristics, outcomes, and outcome measures. As a scoping review, risk-of-bias evaluation and GRADE assessment were not required^23^; however, critical appraisal of SRMA evidence was performed using the AMSTAR-2 checklist and the resulting appraisal supported the assessment of evidence.^24^

To address the secondary aim, implementation strategies in the RCTs were retrospectively coded.^25^ One implementation science framework (Theoretical Domains Framework [TDF]^26^) and 2 taxonomies (Behaviour Change Techniques [BCT]^17^ and Expert Recommendations for Implementing Change [ERIC]^27^) were used. Each has been previously used in CRC screening evaluations.^18–20^ The TDF enabled standardization of the drivers of implementation, and BCTs and ERIC allowed us to retrospectively elicit behavior change for implementation. They were used in parallel by 2 reviewers independently, in consultation with an implementation scientist.

## RESULTS

### Literature search

**FIGURE 1:**
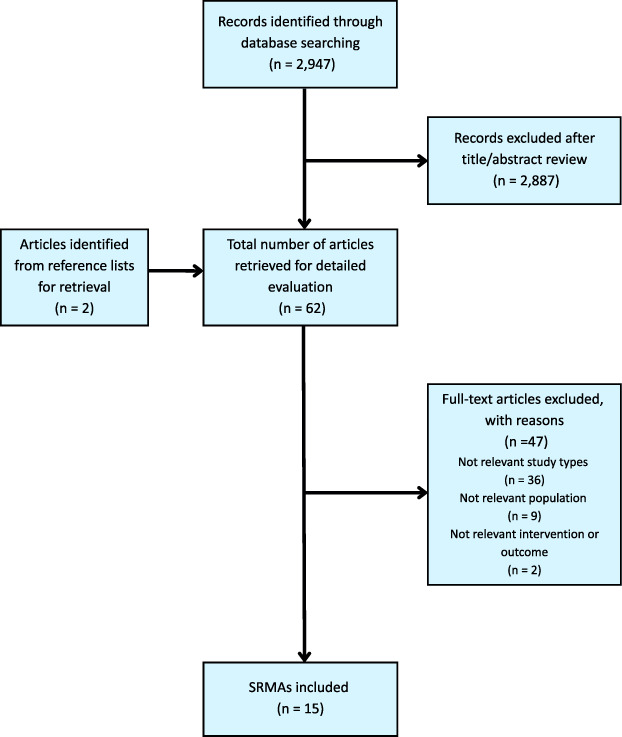
PRISMA flow diagram on systematic reviews with meta-analysis study selection process. Abbreviation: SRMA, systematic review with meta-analysis.

**FIGURE 2:**
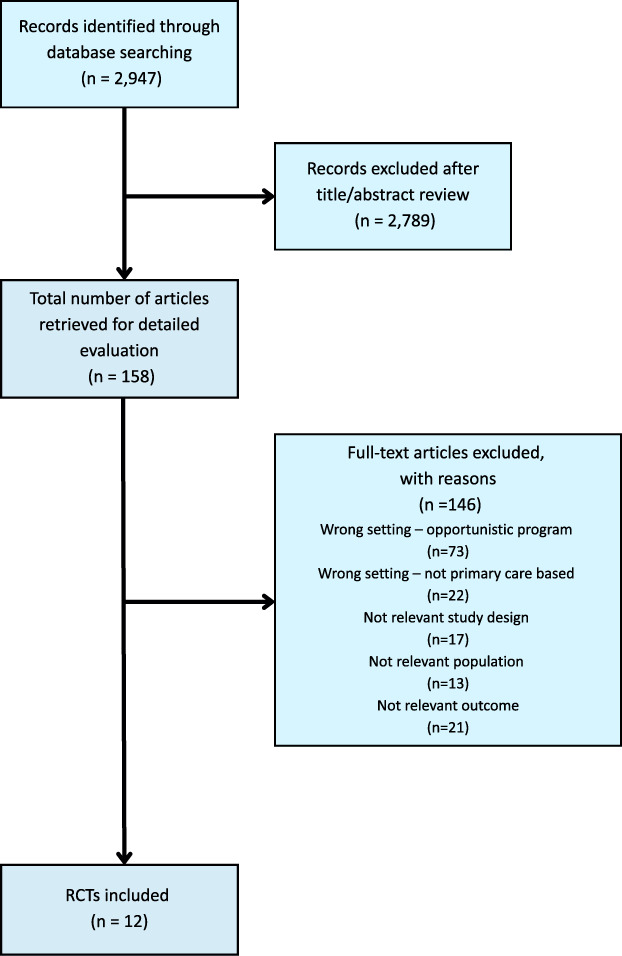
PRISMA flow diagram on randomized controlled trial study selection process. Abbreviation: RCT, randomized controlled trial.

In total, 2947 records were identified. After title, abstract, and full-text screening, 15 SRMAs and 12 RCTs were eligible for inclusion. Figures 1 and 2 show PRISMA flow diagrams.

### Overall characteristics of included articles

In Phase 1, 15 studies were identified.^4,18,28–40^ Most SRMAs included evidence from more than one country (n = 10), with the remaining studies based in the United States (n = 5). Seven SRMAs evaluated a range of interventions,^4,18,29–33^ and 8 focused on either digital interventions,^37^ community-based health worker–led interventions,^34,35^ financial incentives,^36^ motivational interviewing,^38^ telephone reminders,^28^ or decision aids.^39,40^ Six articles provided outcomes specifically on FOBT screening.^4,18,29,31,32,38^ No SRMAs focused on general practice–led interventions. Of the 15 SRMAs, 11 were rated moderate quality and 4 high quality based on the AMSTAR-2 tool.

In Phase 2, the 12 RCTs were conducted in Australia (n = 3), England (n = 3), France (n = 3), Argentina (n = 1), Spain (n = 1), and Canada (n = 1).^3,41–51^ Six studies examined interventions targeted toward GP providers, including printed patient lists,^42,43^ electronic medical record (EMR) alerts,^45^ risk assessment tools,^44^ GP communication skills training,^46^ and quality improvement.^47^ Five studies examined interventions targeted at patients; 4 investigated GP-endorsed reminders^48–51^ and one a risk assessment tool.^41^ Only one RCT evaluated the effectiveness of a multicomponent intervention.^3^

### Relevant SRMA outcomes

Study characteristics and relevant outcomes are detailed in Supplementary Table 6, http://links.lww.com/JPHMP/B673. Relevant outcomes on the uptake of FOBT screening are summarized further and in Table 1 (n = 6).^4,18,29,31,32,38^ FOBT outcomes were generally reported for individual-level interventions and some population-level interventions, but not primary care–level interventions.

**TABLE 1:** Data on the Uptake of Fecal Occult Blood Test Screening in Included Systematic Reviews With Meta-analyses

#	Author(s)	Type of Intervention	Screening Type	Outcome Measure	Estimate (95% CI)	Significance
1	Dougherty et al^29^ (2018)	Kit outreach (mailed)	iFOBT	RR	2.73 (1.60–4.64)	Significant
			gFOBT	RR	1.85 (1.28–2.66)	Significant
		Patient navigation	iFOBT	RR	1.79 (1.32–2.42)	Significant
			gFOBT	RR	2.16 (1.33–3.51)	Significant
		Patient education	iFOBT/gFOBT	RR	1.21 (0.83–1.76)	Not significant
		Patient reminder	iFOBT/gFOBT	RR	1.35 (0.81–2.24)	Not significant
2	Goodwin et al^4^ (2019)	Digital reminder	iFOBT/gFOBT	RR	0.94 (0.83–1.08)	Not significant
		Print materials		RR	0.99 (0.97–1.01)	Not significant
		Behavior priming		RR	1.00 (0.96–1.04)	Not significant
		Advance notification		RR	1.09 (1.07–1.11)	Significant
		Simplified test		RR	1.17 (1.09–1.25)	Significant
		GP endorsement		RR	1.19 (1.10–1.29)	Significant
		Telephone contact		RR	1.23 (1.08–1.40)	Significant
3	Long et al^38^ (2022)	Motivational interviewing	iFOBT/gFOBT	RR—ITT	1.04 (0.96–1.14)	Not significant
				RR—PP	2.43 (1.40–4.21)	Significant
4	Myers et al^31^ (2020)	All interventions combined	iFOBT/gFOBT	RR	1.06 (1.03–1.10)	Significant
		Without added print materials		RR	1.09 (1.05–1.13)	Significant
5	Rubin et al^32^ (2023)	Mail outreach	iFOBT	RR	2.20 (1.74–2.78)	Significant
			FOBT	RR	4.34 (1.29–14.67)	Significant
		Mail outreach with incentive	iFOBT	RR	0.97 (0.81–1.16)	Not significant
		Individualized education	iFOBT	RR	1.07 (0.83–1.38)	Not significant
		Nonindividualized education	iFOBT/FOBT	RR	1.44 (1.07–1.94)	Significant
6	Tsipa et al^18^ (2021)	All interventions combined	iFOBT/gFOBT	OR	1.31 (1.24–1.40)	Significant

Abbreviations: FOBT, fecal occult blood test; gFOBT, guaiac fecal occult blood test; GP, general practitioner; iFOBT, immunochemical fecal occult blood test; ITT, intention-to-treat; OR, odds ratio; PP, per-protocol; RR, risk ratio

#### Population-level interventions

SRMA evidence revealed that 2 population-level interventions—kit outreach or proactive distribution of kits and simplified test procedures—led to significant increases in FOBT screening participation.^4,29,32^ Specifically, kit outreach showed a large relative increase in iFOBT and gFOBT participation among average-risk adults, with RR 2.73 (95% CI 1.60–4.64, *P* < .001, I^2^ = 81.4%) and RR 1.85 (95% CI 1.28–2.66, *P* < .001, I^2^ = 95%), respectively,^29^ as well as in low-income populations for iFOBT and gFOBT, with RR 2.20 (95% CI 1.74–2.78, *P* < .005) and RR 4.34 (95% CI 1.29–14.67, *P* < .005), respectively.^32^ Simplified test procedures—such as offering iFOBT instead of gFOBT—also increased FOBT participation to a lesser extent (RR 1.17 [95% CI 1.09–1.25], I^2^ = 98.5%).^4^

#### Individual-level interventions

##### Patient navigation

One United States–based study reported that patient navigation—supported guidance by a general practice staff member (eg, phone consult with a nurse to explain how to use kit)—led to a significant increase in both iFOBT (RR 1.79 [95% CI 1.32–2.42]) and gFOBT screening participation (RR 2.16 [95% CI 1.33–3.51], *P* < .001, I^2^ = 94.8%).^29^

##### Patient education

In United States–based studies, patient education (providing patients with information and resources) had a nonsignificant positive impact on FOBT screening increase (RR 1.21 [95% CI 0.83–1.76], *P* < .01, I^2^ = 77.1%)^29^ and for low-income adults when the education was personalized based on patient needs (RR 1.07 [95% CI 0.83–1.38], *P* > .005, I^2^ = 87%).^32^ However, the same study found a small significant impact of nonindividualized patient education for low-income adults (RR 1.44 [95% CI 1.07–1.94], *P* < .005, I^2^ = 85%).^32^

##### Patient reminders

Two SRMAs investigated patient reminders.^4,29^ The effect on FOBT kit return varied for advance notification (RR 1.09 [95% CI 1.07–1.11], I^2^ = 1.1%), GP endorsement (RR 1.19 [95% CI 1.10–1.29] I^2^ = 95.6%), and telephone contact (RR 1.23 [95% CI 1.08–1.40] I^2^ = 61.5%).^4^ However, no statistically significant increase was observed in the overall population for digital reminders (RR 0.94 [95% CI 0.83–1.08], I^2^ = 53.9%).^4^ The second SRMA also reported a nonsignificant increase in FOBT uptake as a result of all patient reminders (RR 1.35 [95% CI 0.81–2.24], *P* = .15, I^2^ = 43%).^29^

##### Other interventions

Motivational interviewing (person-centered counseling approach) led to a significant increase in participation using a per-protocol (PP) analysis (RR 2.43 [95% CI 1.40–4.21], I^2^ = 98.9%), but not using intention-to-treat (ITT) analysis (RR 1.04 [95% CI 0.96–1.14], I^2^ = 82%).^38^

Other individual-level interventions assessed were not statistically significant. These included behavior priming (eg, text modifications in printed materials distributed alongside kit; RR 1.00 [95% CI 0.96–1.04], I^2^ = 85.5%) and added printed materials to standard mail-out (RR 0.99 [95% CI 0.97–1.01], I^2^ = 85.5%).^4^

#### Multicomponent interventions

Multicomponent interventions or combined effects of single interventions were assessed. Tsipa et al.^18^ combined the effects of interventions from 56 studies, reporting a significant increase in FOBT with an odds ratio of 1.31 (95% CI: 1.24–1.40, I^2^ = 97.1, *P* < .001). Myers et al.^31^ found that multicomponent interventions increased FOBT screening compared with single-component interventions (RR 1.06 [95% CI 1.03–1.10] I^2^ = 86.1%), which increased with the exclusion of printed materials (RR 1.09 [95% CI 1.05–1.13]).

### Relevant RCT outcomes

RCT study outcomes on uptake of FOBT are listed in Table 2 (additional information in Supplementary Table 7, http://links.lww.com/JPHMP/B673). None included population-level interventions.

**TABLE 2:** Data on the Uptake of Fecal Occult Blood Test Screening in Included Randomized Controlled Trials

#	Author(s)	Type of Intervention	Outcome Measure	Estimate (95% CI)	Significance
1	Aubin-Auger et al^46^ (2016)	GP 4-hr educational training session	Participation rate	UC: 24.5% INT: 36.7%	Significant
2	Cross et al^49^ (2021)	GP-endorsed invitation letter with gFOBT kit	OR—PP	1.03 (1.01–1.05) UC: 58.7% INT: 59.4%	Significant
3	Dodd et al^3^ (2019)	Multicomponent intervention	OR	10.24 (2.9–36.6) UC: 6.0% INT: 39.0%	Significant
4	Emery et al^44^ (2023)	Decision support tool for GPs	OR	1.36 (0.99–1.86)	Not significant
			OR—those due for screening only	2.31 (1.51–3.53) UC: 38.9% INT: 59.8%	Significant
5	Guiriguet et al^45^ (2016)	Electronic reminder alert to GP in primary care EMR	OR—ITT	1.08 (0.97–1.20) UC: 42.2% INT: 44.1%	Not significant
			OR—PP	OR 1.09 (0.99–1.19) UC: NR INT: NR	Not significant
			Adjusted OR—PP	1.11 (1.02–1.22) UC: NR INT: NR	Significant
6	Hirst et al^51^ (2017)	GP-endorsed text message reminder	OR—first timer invitees	1.29 (1.04–1.58) UC: 34.9% INT: 40.5%	Significant
7	Irazola et al^47^ (2023)	Quality improvement strategies with PDSA cycles	OR	2.5 (1.4–4.4) UC: 54.2% INT: 75%	Significant
8	Kiran et al^48^ (2018)	GP-endorsed reminder letter or phone call	Absolute difference—ITT—female	6.1% (1.4–10.8) INT 1: 20.2% INT 2: 26.3%	Significant
			Absolute difference—ITT—male	3.7% (0.5–7.9) INT 1: 21.1% INT 2: 24.8%	Not significant
			Absolute difference—PP—female	6.4% (0.9–11.8) INT 1: 14.9% INT 2: 21.2%	Not significant
			Absolute difference—PP—male	5.1% (0.2–9.9) INT 1: 16.3 INT 2: 21.4	Not significant
9	Le Breton et al^42^ (2016)	A computer-generated print list for GPs for patients not up to date with CRC screening	Adjusted RR—ITT	1.07 (0.95–1.20) UC: 31.2% INT: 32.9%	Not significant
10	Raine et al^50^ (2016)	GP endorsed initial invitation letter	Adjusted OR—ITT	1.07 (1.04–1.10) UC: 57.5% INT: 58.2%	Significant
11	Rat et al^43^ (2017)	Printed list for GPs of patients not up to date with CRC screening	OR—ITT—patient-specific reminder	1.27 (1.15–1.41) UC: 18.7% INT 1: 22.6%	Significant
			Generic reminder	1.09 (0.98–1.21) UC: 18.7% INT 2: 20.3%	Not significant
12	Trevena et al^41^ (2022)	Risk assessment tool for patients	Adjusted OR—PP	1.66 (1.25–2.22) UC: 15.1% INT: 24.9%	Significant

Abbreviations: CRC, colorectal cancer; EMR, electronic medical record; GP, general practitioner; gFOBT, guaiac fecal occult blood test; ITT, intention-to-treat; INT, intervention; NR, not reported; OR, odds ratio; PDSA, Plan-Do-Study-Act; PP, per-protocol; RR, risk ratio; UC, usual care.

#### Primary care–level interventions

The primary care-level interventions identified in this study were in the general practice setting.

##### GP risk assessment tools

One Australian RCT evaluated the effectiveness of a risk assessment tool designed to be completed by GPs in Melbourne.^44^ The “Colorectal cancer RISk Prediction” (CRISP) tool was implemented over 12 months and led to a 20.3% absolute increase (OR 2.31, 95% CI = 1.51–3.53, *P* < .001) in FOBT screening among those due for screening at average risk of CRC.^44^

##### Provider reminders

Providing GPs with lists of overdue patients had mixed results with no evidence of an increase in FOBT screening in Paris, France (adjusted relative risk 1.07, 95% CI 0.95–1.20), *P* = .27),^42^ but an increase in iFOBT screening in a trial in the Loire-Atlantique and Vendée areas of France (OR 1.27, 95% CI = 1.15–1.41, *P* < .001).^43^ Guiriguet et al^45^ trialed an EMR reminder in Barcelona, Spain, which did not significantly increase participation in ITT analysis (44.1% vs. 42.2%, intervention vs. control; OR 1.08 [95% CI 0.97–1.20], *P* = .146). However, an adjusted PP analysis found a small, significant effect size (adjusted OR 1.11 [95% CI 1.02–1.22], *P* = .02).

##### Provider education

GP communication skills training was found to significantly increase gFOBT screening participation in the Val d’Oise region in France, from 24.5% (±10.1%) in the usual care group to 36.7% (±20.3%) in the intervention group (*P* = .03).^46^

##### Quality improvement

Irazola et al^47^ evaluated the effectiveness of a quality improvement Plan-Do-Study-Act (PDSA)–focused intervention in primary care in Mendoza province, Argentina. The study had a significantly positive effective in increasing iFOBT screening, with an OR of 2.5 (95% CI 1.4–4.4, *P* < .01).

#### Individual-level interventions

##### Patient risk assessment tools

One Australian RCT evaluated the effectiveness of a CRC risk assessment tool designed to be completed by patients.^41^ The “Which test is best” tool showed an FOBT uptake difference of 15.1% in the usual care to 24.9% in the intervention (adjusted OR 1.66 [95% CI 1.24–2.22], *P* = .001) in a PP analysis.^41^

##### Patient reminders

Four RCTs evaluated the effectiveness of patient reminders.^48–51^ A United Kingdom–based study found that a GP-endorsed text message reminder was effective at increasing first-time gFOBT screening participation in London (OR 1.29 [95% CI 1.04–1.58], *P* = .02).^51^ However, the intervention did not have a statistically significant impact on the gFOBT screening participation rate for participants who had previously been screened in the organized program (OR 0.98 [95% CI 0.89–1.08], *P* = .66).

Two earlier UK trials found that GP-endorsed initial invitation letters increased gFOBT participation.^49,50^ In the study by Raine et al,^50^ the participation rate increased from 57.5% in usual care to 58.2% in the intervention group using ITT analysis (adjusted OR 1.07 [95% CI 1.04–1.10], *P* < .001). In the study by Cross et al,^49^ the participation rate increased from 58.7% to 59.4% using PP analysis (OR 1.03 [95% CI 1.01–1.05], *P* < .001).

Among patients who were overdue for screening in Toronto, Canada, a phone call from a trained general practice staff member increased FOBT uptake compared with a mailed reminder letter for women using ITT analysis (absolute difference 6.1% [95% CI 1.4–10.8], *P* = .01).^48^ Despite an absolute increase of 3.7%, the result was not significant for men.

#### Multicomponent interventions

One Australian RCT adopted a multicomponent intervention, evaluating the effectiveness of point-of-care provision of iFOBT kits (population-level), in-person GP endorsement of iFOBT screening to patients during their appointment (primary care–level), and patient education (individual-level) in New South Wales.^3^ The study found that the rate of screening participation increased from 6.0% in usual care to 39.0% in the intervention group (OR 10.24 [95% CI 2.9–36.6], *P* < .01).^3^

### Implementation barriers and facilitators and strategies

Key barriers were mapped to 4 domains of the TDF: Knowledge, Environmental Context & Resources, Skills and Memory, Attention & Decision Process (Supplementary Table 8, http://links.lww.com/JPHMP/B673). Identified barriers included the following: limited consultation time for providing CRC screening advice,^3,42,43,46^ no formal involvement of GPs in population-based screening programs,^47,48,50^ lack of access to test kits,^3,44,47^ not patient-centered communication style,^42,46^ patient lack of awareness of their CRC risk,^41^ GP lack of awareness of CRC screening recommendations,^41^ underutilization of EMR reminders,^45^ performance issues with EMR reminders,^3^ suboptimal family history records in general practice,^41^ lack of data and resources for generating lists of nonparticipants,^43^ insufficient GP training,^42^ patient forgetfulness,^51^ and provider forgetfulness.^42^

Fifteen BCTs were identified (Supplementary Table 9, http://links.lww.com/JPHMP/B673). BCTs targeted at providers included the following: prompts/cues,^42–46^ instruction on how to perform the behavior,^43,45,46^ problem solving,^44,47^ credible source,^44,45^ restructuring the physical environment,^44,47^ goal setting (behavior),^47^ goal setting (outcome),^47^ social support,^47^ information about antecedents,^44^ information about health consequences,^45^ demonstration of the behavior,^46^ social comparison,^46^ information about others' approval,^46^ behavioral practice/rehearsal,^46^ and adding objects to the environment.^44^ BCTs targeted at patients included the following: credible source,^3,41,48–51^ prompts/cues,^3,41,48,49,51^ information about health consequences,^3,41,48^ instruction on how to perform the behavior,^3^ and adding objects to the environment.^3^

Sixteen strategies were retrospectively coded using the ERIC taxonomy (Supplementary Table 10, http://links.lww.com/JPHMP/B673). Identified strategies included the following: distribute educational materials,^3,41,45,46,48–51^ develop educational materials,^3,41,44,46–48^ increase demand,^3,48–51^ identify and prepare champions,^42,43,45,46^ prepare patients/consumers to be active participants,^3,41,48^ remind clinicians,^41–46^ practice facilitation,^41,44,47^ develop and implement tools for quality monitoring,^44,47^ develop and organize quality monitoring systems,^44,47^ engage or include patients/consumers and families in the implementation effort,^44,46^ alter incentive allowance structure,^41^ change physical structure and equipment,^3^ conduct educational outreach visits,^46^ obtain and use patient/consumer and family feedback,^41^ intervene with patients/consumers to enhance uptake and adherence,^44^ and unclassified.^48^

## DISCUSSION

This scoping review identified 15 SRMAs and 12 RCTs, which were evaluated to understand the effectiveness of interventions to increase CRC screening participation. The study identified implementation strategies addressing 13 TDF barriers, 15 BCTs, and 16 ERIC strategies.

### Population-level interventions

Kit outreach is a key intervention for improving CRC screening participation, supported by high-quality SRMA evidence.^29,32^ Kit outreach is often targeted toward hard-to-reach populations to combat structural access barriers. For example, national Australian trial data showed that active kit distribution through primary health care centers in addition to the standard mailed kit increased CRC screening participation from 23.3% to 39.8% for Indigenous Australians.^52^ Other population-level interventions, such as mass media campaigns that include primary care promotion activities, can be effective methods for increasing participation^53^ but were not included in this review.

### Primary care–level interventions

RCT evidence indicates that use of CRC risk assessment tools by practice staff to identify patients eligible for CRC population screening may be an effective mechanism for increasing participation.^44^ Importantly, tools can initiate discussion with a GP and prompt kit demonstrations and endorsement, and thus, their isolated effectiveness is not clear. A systematic review of RCTs, not specific to CRC screening, indicated that cancer risk assessment tools in primary care may improve knowledge but, alone, may not change screening behavior.^54^ Further research is warranted to understand their effectiveness, particularly a multicomponent approach that involves risk assessment and subsequent interventions at improving risk-appropriate screening.

GP reminders of patients overdue for CRC screening have inconsistent effects based on RCT evidence.^42,43,45^ Printed patient lists showed mixed results.^42,43^ However, EMR reminders were moderately effective.^45^ A survey of health care providers in Nebraska, United States, found that computerized reminder systems may enhance patients' awareness of their CRC screening status, although successful implementation required infrastructure support (eg, data registries to maintain accurate data records).^55^ EMR reminders are low-cost and can be integrated into the routine workflow of general practice.^45^ However, further trial data are needed to assess their effectiveness and to identify implementation strategies required for practices to transition from paper-based to electronic record-keeping.

The World Health Organization recognizes quality improvement as an essential component of primary care,^56^ with models such as the PDSA widely used. Our review identified an effective PDSA-based primary care–level intervention.^47^

No trial data were identified related to data cleansing, practice system, or software enhancement, possibly because evaluations are not always publicly reported or are undertaken opportunistically.^57^

### Individual-level interventions

Patient navigation led to significant improvements in CRC screening in a high-quality SRMA.^29^ Tailoring of education to patients' needs was not deemed to be effective in average-risk adults.^29^ However, delivery of more generalized, nonindividualized CRC education may be effective for low-income adults,^32^ although results were heterogeneous.

RCT evidence also indicated that risk assessment tools completed by patients before a GP visit may be effective.^41^ However, subgroup analyses showed that risk-appropriate screening increased significantly for those at high CRC risk, but not those at average or moderate risk.^41^ Further research is warranted to understand the effectiveness of such tools for various at-risk populations.

Considerable evidence was found on the effectiveness of patient reminders.^4,29,48–51^ Live 30-day reminder telephone calls were associated with the largest increase in CRC screening participation of any intervention type.^4^ Similarly, phone calls from a trained GP were more effective than mailed GP-endorsed reminders, albeit more costly.^48^ Only one study evaluated the effectiveness of a text message reminder alone, reporting an increase in CRC screening participation among never-screeners but no significant effect on those previously screened.^51^ An Australian trial not captured in this review found that a text message reminder increased kit return in 50–60-year-old patients^58^ and is being trialed more widely.^59^ These findings suggest the importance of careful consideration of target population and format delivery in the potential effectiveness of a patient reminder intervention.

### Multicomponent interventions

Both SRMA and RCT evidence support the effectiveness of multicomponent interventions for increasing CRC screening participation.^3,18,31^ One multicomponent RCT involving a combination of interventions resulted in significant improvements in CRC screening participation, with patients attending these practices up to 10 times more likely to complete screening in comparison with usual care.^3^ The authors suggested that the RCT's success may be due to its multicomponent design, which addressed key barriers. For instance, providing patients with printed information to read before their consultation helped overcome the barrier of limited consultation time.^3^ Multicomponent interventions can also effectively target hard-to-reach populations—for example, a process and outcomes evaluation of a community health–led program in El Paso County, Texas, revealed that patient education, navigation, and no-cost screening combined successfully improved FOBT uptake in a largely uninsured Hispanic population.^60^ Larger trials should be conducted to confirm these results.

### Behavior change and implementation

Changing behavior can be challenging; success is more likely when interventions are grounded in evidence-based behavior change. No RCTs explicitly reported implementation barriers and facilitators or strategies and thus were coded retrospectively. Given that a limited number of TDF domains, BCTs, and ERIC strategies were identified, no conclusions have been drawn about their application. Future research should further explore the alignment between the TDF domains, BCTs, and ERIC strategies,^61,62^ and trials should explicitly report on implementation approaches used to facilitate adaptation and sustainability.

### Strengths and limitations

This study evaluated SRMA evidence on intervention effectiveness on CRC screening participation, with a detailed review of RCT evidence on general practice–led interventions. Key strengths of this review include the quality of the evidence and the iterative design, which allowed the scope of the search to change based on the previous phase findings. The study adapted a published search strategy, performed quality assessments of the included systematic reviews using the AMSTAR-2 checklist, coded the included RCTs to one implementation science framework and 2 taxonomies, and undertook a search of internationally published literature to capture existing and emerging interventions.

This scoping review has several limitations. The review only captured more recent evidence, excluding studies published earlier than May 2016 as a continuation of the last systematic review available.^18^ Phase 1 did not include a formal overlap assessment of SRMAs, which may have led to overestimation of evidence quantity and strength. Furthermore, many of the included SRMAs reported high I^2^ values, reflecting considerable heterogeneity. Phase 2 excluded observational, nonrandomized, and implementation research study designs. The focus was on studies reporting FOBT screening results only; applicable interventions to increase uptake of other screening modalities or cancer types were excluded. Studies targeting high-risk populations were also excluded, potentially omitting useful insights. This review may be subject to publication bias, as only published studies were included, and language bias, as only English-language articles were reviewed.

**Implications for Policy and Practice**

■ General practice is a key setting for improving CRC screening participation, which remains suboptimal in organized programs worldwide. Policymakers and public health practitioners should target and work with general practice to increase CRC screening uptake.
■ Evidence-based, general practice–led interventions that show potential to increase CRC screening uptake include patient reminders, risk assessment tools, and kit outreach. Policymakers and public health practitioners could further encourage general practice by supporting the implementation of evidence-based interventions to boost CRC screening uptake.
■ Evidence for multicomponent interventions that combine intervention strategies across the individual, primary care, and population level is promising but limited; policymakers could fund further trials to explore their effectiveness.
■ Policymakers and public health practitioners could facilitate intervention implementation by drawing on evidence-based implementation successes applicable to general practice.

## Supplementary Material
