# Supplementary material for: Effective General Practice–Led Interventions to Increase Uptake in Colorectal Cancer Screening: A Scoping Review
Source: J Public Health Manag Pract. 2026 May 8;32(4):437–48. doi: 10.1097/PHH.0000000000002367 (PMC13178772; doi:10.1097/PHH.0000000000002367)
Supplement: Supplementary file 1 [file phh-32-437-s001.docx]

**Effective general practice-led interventions to increase uptake in colorectal cancer screening: a scoping review**

**Supplementary Tables**

S1. PICO search for systematic reviews with meta-analyses

S2. PICO search for randomised controlled trials

S3. Search strategy

S4. Selection criteria for systematic reviews with meta-analyses

S5. Selection criteria for randomised controlled trials

S6. Characteristics of included systematic reviews with meta-analyses

S7. Characteristics of included randomised controlled trials

S8. Coding of barriers to the Theoretical Domains Framework

S9. Coding of components to the Behaviour Change Technique taxonomy

S10. Coding of strategies to Expert Recommendations for Implementing Change framework

**S1. PICO search for systematic reviews with meta-analyses**

| **Population** | **Intervention** | **Comparison** | **Outcome** | **Study design** |
| --- | --- | --- | --- | --- |
| Adult population, eligible for CRC screening and at average risk of CRC | Any intervention designed to increase participation in primary CRC screening | Comparator intervention, control group or usual care | Uptake of primary CRC screening | SRMAs |
| *SRMA = systematic review with meta-analyses; CRC= colorectal cancer; RCT; randomised controlled trial* | | | | |

**S2. PICO search for randomised controlled trials**

| **Population** | **Intervention** | **Comparison** | **Outcome** | **Study design** |
| --- | --- | --- | --- | --- |
| Adult population, eligible for CRC screening and at average risk of CRC | Intervention designed to increase participation in iFOBT or gFOBT, as part of organised screening program, delivered in general practice setting | Comparator intervention, control group or usual care | Uptake of iFOBT or gFOBT screening | RCTs |
| *CRC= colorectal cancer, iFOBT= immunochemical faecal occult blood test, gFOBT=guaiac faecal occult blood test, RCT; randomised controlled trial* | | | | |

**S3. Search Strategy**

| **#** | **Search term** | **# Results**  **May 2016 – Jun 2022** | **# Results**  **Aug 2022 – Aug 2023** |
| --- | --- | --- | --- |
| **Step 1: Intervention** | | | |
| 1 | intervention study/ | 591068 | 601,733 |
| 2 | intervention*.tw. | 13 | 3,664,422 |
| 3 | health promotion/ | 212642 | 221,730 |
| 4 | patient navigat$.tw. | 4098 | 4,673 |
| 5 | reminder$.mp. | 48284 | 52,490 |
| 6 | incentive$.mp. | 105751 | 114,046 |
| 7 | reduc$ cost.mp. | 11049 | 12,023 |
| 8 | mass medium/ | 20354 | 21,132 |
| 9 | attitude to health/ | 212251 | 218,746 |
| 10 | health behavior/ | 160076 | 168,372 |
| 11 | health belief/ | 14133 | 15,760 |
| 12 | health belief model/ | 2701 | 3,298 |
| 13 | risk reduction/ | 133625 | 144,482 |
| 14 | behavio?r change/ | 57688 | 64,218 |
| 15 | Social Cognitive Theory/ | 16153 | 16,507 |
| 16 | persuasive communication/ | 18850 | 19,317 |
| 17 | "theory of planned behavior"/ | 5017 | 5,832 |
| 18 | message fram*.mp. | 1902 | 2,138 |
| 19 | implementation intention$.mp. | 1932 | 2,095 |
| 20 | protection motivation theory.mp. | 1366 | 1,606 |
| 21 | social cognition model$.mp. | 304 | 316 |
| 22 | self efficacy.mp. | 142610 | 157,976 |
| **23** | **or/ 1-22** | 1637634 | 5,043,132 |
| **Step 2: Type of Cancer** | | | |
| 24 | colorectal cancer/ | 272591 | 299,347 |
| 25 | bowel cancer.mp. | 6454 | 6,835 |
| 26 | colon cancer/ | 153924 | 163,012 |
| 27 | rectum cancer/ | 86296 | 92,783 |
| 28 | (colorectal adj2 neoplasm$).mp. | 119289 | 128,021 |
| **29** | **or/24-28** | 491839 | 531,181 |
| **Step 3: Outcomes** | | | |
| 30 | cancer screening/ | 128306 | 140,827 |
| 31 | self examination/ | 5337 | 5,546 |
| 32 | cancer prevention/ | 46105 | 48,989 |
| 33 | health education/ | 186713 | 194,236 |
| 34 | health literacy/ | 27741 | 32,675 |
| 35 | health promotion/ | 212642 | 221,730 |
| 36 | health knowledge.mp. | 166484 | 172,000 |
| 37 | early diagnosis/ | 157751 | 167,500 |
| 38 | patient compliance/ | 204289 | 213,175 |
| 39 | patient adherence.mp. | 12827 | 14,022 |
| 40 | health care access/ | 77741 | 89,020 |
| 41 | barium enema.mp. | 17189 | 17,418 |
| 42 | endoscop$.mp. | 772888 | 830,962 |
| 43 | f?ecal occult blood.mp. | 11526 | 12,036 |
| 44 | occult blood/ | 16805 | 17,654 |
| 45 | occult blood test/ | 6418 | 7,344 |
| 46 | occult blood.mp. | 27366 | 29,393 |
| **47** | **or/30-46** | 1904657 | 2,030,423 |
| **Step 4: limits** | | | |
| 48 | 23 and 29 and 47 | 5971 | 12,361 |
| 49 | Remove duplicates from 48 | 5154 | 1,334 |
| 50 | Limit 49 to yr=”2016 -Current” | 1677 | 1,315 |
| 51 | Limit 50 to English language | 1652 | 1,301 |
| 52 | Limit 51 to human | 1640 | 655 |
| 53 | limit 52 to conference abstracts [Limit not valid in Ovid MEDLINE(R),Ovid MEDLINE(R) Daily Update,Ovid MEDLINE(R) PubMed not MEDLINE,Ovid MEDLINE(R) In-Process,Ovid MEDLINE(R) Publisher,APA PsycInfo; records were retained] | 659 | 346 |
| 54 | Limt 53 to medline |  | 309 |
| 55 | 53 not 54 |  | 992 |
| 56 | 52 not 55 | 2139 | 808 |
| *Database search using Ovid (Embase+PsycInfo+Medline) 2016 to 16^th^ June 2022. = MeSH term, */$ = Truncation, .tw = title, abstract, .mp = title, abstract, subject, heading, Exp = explode search, ? = letter may appear or not* | | | |

**S4. Selection criteria for systematic reviews with meta-analyses**

| **Selection criteria** | **Inclusion** | **Exclusion** |
| --- | --- | --- |
| **Study type** | Intervention | Observational |
| **Study design** | Systematic reviews with meta-analyses | Systematic reviews without meta-analysis  RCTs  Non-randomised trials  Quasi-experimental studies  Pre- and post-studies  Cohort or case-control studies  Case series  Case report  Review (not systematic) |
| **Population** | Adults ≥18 years, average risk population | Above average or high-risk population.  This included people with a personal or family history of CRC, genetic predisposition, as well as those who are part of an ethnic group known to have elevated risk of CRC |
| **Intervention** | Intervention designed to increase to uptake of primary CRC screening including endoscopic (colonoscopy, flexible sigmoidoscopy) and non-endoscopic (iFOBT, gFOBT) screening. | No intervention or intervention designed to increase follow-up of a positive primary screen (e.g., diagnostic colonoscopy) |
| **Comparator** | Comparator intervention, control group or usual care | No comparator |
| **Outcome** | *Uptake of CRC screening:*  Reported quantitatively as odds or risk ratios with 95% confidence interval (CI) or raw, intention-to-treat uptake data (% screened). | No uptake data provided  Return rate of CRC screening not reported separately to other types of cancer screening (i.e., breast, cervical, lung or prostate screening) |
| **Publication date** | Studies published from May 2016 onwards | Pre May 2016 |
| **Publication type** | Original journal article in full | Conference abstracts that do not report all data  Posters |
| **Language** | English | Not in English |
| *CI= confidence interval, CRC= colorectal screening, iFOBT= immunochemical faecal occult blood test, gFOBT=guaiac faecal occult blood test, RCTs = randomised controlled trials* | | |

**S5. Selection criteria for randomised controlled trials**

| **Selection criteria** | **Inclusion** | **Exclusion** |
| --- | --- | --- |
| **Study type** | Intervention | Observational |
| **Study design** | RCT | Non-randomised trials  Quasi-experimental studies  Pre- and post-studies  Cohort or case-control studies Case series  Case report  Review |
| **Population** | Adults ≥18 years, average risk population | Above average or high-risk population.  This includes people with a family history of bowel cancer, genetic predisposition, as well as those who are part of an ethnic group known to have elevated risk of CRC |
| **Intervention** | Intervention designed to increase participation in organised population-based iFOBT or gFOBT screening program delivered in a primary care, general practice-based setting. Based in a country with a similar organised population-based screening program to Australia. | Not an implementation intervention (e.g., a technical intervention or programmatic change)  Aimed at increasing uptake of endoscopic CRC screening including colonoscopy, flexible sigmoidoscopy or emerging forms of CRC screening not readily available in Australia (e.g., mt-sDNA) |
| **Comparator** | Comparator intervention, control group or usual care | No comparator |
| **Outcome** | *Uptake of gFOBT/iFOBT screening:*  Reported as risk ratios (95% CI) or raw, intention-to-treat uptake data (% screened). | No uptake data provided  Return rate of non-endoscopic screening not reported separately to other types of CRC screening (i.e., colonoscopy) |
| **Publication date** | Studies published from 2016 onwards | August 2023 |
| **Publication type** | Original journal article in full. | Conference abstracts that did not report all data  Posters |
| **Language** | English | Not in English |
| *CI= confidence interval, CRC= colorectal cancer screening, iFOBT= immunochemical faecal occult blood test, gFOBT=guaiac faecal occult blood test, RCTs = randomised controlled trials* | | |

**S6. Characteristics of included systematic reviews with meta-analyses**

| **#** | **Author(s)** | **Type of studies included** | **Intervention** | **Target of intervention** | **Outcome** |
| --- | --- | --- | --- | --- | --- |
| 1. | Bellhouse et al. (2017) (1) | RCT | Intervention facilitated by a community-based health worker that does not require formal education or qualifications to fulfill the role. The role of the worker was to provide initial patient engagement with health care professionals and access to cancer screening.    Examples of included intervention components: education information, educational pamphlet, educational brochure, flipchart, logistical assistance, barriers counselling, and follow-up reminders. | Patient | Results showed that participation in community-based health worker interventions was associated with increased screening for breast, cervical, and bowel. |
| 2. | Dougherty et al. (2018) (2) | RCT | Any intervention design to facilitate CRC screening uptake in the US.    Examples of included interventions: kit outreach, patient navigation, patient education, patient reminder, patient financial incentive, clinician reminder, academic detailing, multicomponent | Patient or Provider | Results found multiple interventions were effective in increasing uptake for CRC screening in diverse populations in the US. |
| 3. | Facciorusso et al. (2021) (3) | RCT | Intervention in the form of a financial incentive targeted towards patients in the US.    Examples of financial incentives: fixed, lottery-based, less than $5, greater than $10, with mailed kit outreach, with mailed or electronic reminder only | Patient | Results found that adding financial incentives is associated with a small benefit of increasing uptake in CRC screening. |
| 4. | Goodwin et al. (2019) (4) | Quantitative studies | Any intervention to facilitate mail-out FOBT uptake.    Examples of included interventions: telephone contact, GP endorsement, simplified test procedure, advance notification, added print materials, and digital reminder. | Patient | Results found statistical significance in the four key intervention strategies: advance notification, GP endorsement, telephone contact, and the simplification of testing procedures. The highest average increase in the rate of kit was linked to interventions involving telephone contact. |
| 5. | Jager et al. (2019) (5) | RCT | Mailed kit outreach intervention targeted towards patients not-up-to date with CRC screening in the US.    An additional meta-analysis with and without telephone reminder was conducted by the authors. | Patient | Mail outreach with gFOBT or iFOBT was associated with a large and consistent increase in CRC screening completion. |
| 6. | Lau et al. (2022) (6) | RCT | Digital interventions likely to promote CRC screening uptake.    Examples of included interventions: digital technology interventions such as decision-making aids and tailored educational interventions. | Patient | Digital decision-making aids significantly improved CRC screening uptake compared to tailored digital educational interventions and usual care. |
| 7. | Long et al. (2022) (7) | RCT | Motivational interviewing intervention    Defined as a collaborative person-centered counselling approach. Practitioners establish rapport with patients to explore their motivation or ambivalence for screening, Motivational interviewing focuses on minimising resistance and building self-efficacy instead of having a confrontation or persuasion. | Patient | Results show that motivational interviewing was statistically significant in increasing CRC screening in both intention-to-treat and per-protocol analysis |
| 8. | Myers et al. (2020) (8) | Quantitative studies | Any intervention designed to increase mail-out FOBT uptake.    There were seven distinct types of interventions including: advance notification, GP endorsement, simplified test, added print materials, collection paper, financial incentive, and community drop-off location. | Patient | Results showed nonsignificant results in intervention effects between subpopulations however combining interventions led to increased participation. |
| 9. | Posadzki et al. (2016) (9) | RCT | Automated telephone reminder system as part of multicomponent intervention.    Examples of multicomponent intervention included: letters, prompts for patients and clinicians, and provision of testing kits. | Patient or Provider | Results show that for prevention, multimodal automated telephone communication systems increase the number of people screening for CRC compared with control.    Interactive Voice Response systems probably increased screened numbers for CRC up to six months. |
| 10. | Ramli et al. (2021) (10) | RCT; non-randomised trial | E-media decision aid intervention designed to facilitate CRC screening uptake.    Examples of the content of the e-media decision aids include: a series of questions regarding health, prior screening, and readiness to screen, video of patient-doctor conversation, individualised reminder message, information about importance and ease of screening, patient experiences of follow-up test, culturally tailored information, constructs of preventative health model, and overview of test options. | Patient | CRC-screening promotion via e-media can potentially increase screening participation in primary healthcare settings. |
| 11. | Rana et al. (2023) (11) | RCT | Effectiveness of community health worker-led interventions in improving the CRC screening uptake rate in racial and ethnic minority populations.    Examples of included intervention components: multicomponent interventions (a combination of educational sessions and/or mailed letter and/or phone calls and/or navigational services) and single-component interventions (educational sessions or phone calls or navigational services) | Provider | Results show that CRC screening uptake was improved in participants who received community health worker-led interventions compared to participants with those who received no intervention. |
| 12. | Rubin et al. (2023) (12) | RCT | Interventions to increase CRC screening adherence in low-income settings in the US.    Examples of included intervention components: mailed outreach, patient navigation, patient education, reminder types and other comparisons (academic detailing and tailored telephone education) | Patient | Results showed that mail outreach and patient navigation are the most effective strategies to increase CRC screening in low-income populations. |
| 13. | Tsipa et al. (2021) (13) | RCT | Any intervention designed to facilitate an increase in CRC screening.    Examples of included interventions delivered are face-to-face, remote, mixed, delivered one-on-one or to a group, by clinically trained health professionals, non-clinically trained health professionals, by research staff or non-person dependent, electronic or paper-based media, in community or primary care locations, and in free or not free healthcare settings. | Patient or Provider | Health professionals led-interventions were significantly more effective at improving screening uptake rates compared to those provided by those that were not person-dependent. |
| 14. | Volk et al. (2016) (14) | RCT | Decision aid interventions, for example e-media and paper-based.  Any intervention that provides information about pros and cons of at least two screening options (including no screening) and allowed a user to consider the trade-offs between options. | Patient | Results showed no significance in screening interest or behaviour despite decision aid patient having greater knowledge than patients who received general CRC screening information. |
| 15. | Yakoubovitch et al. (2023) (15) | RCT | The effect of behavioural interventions on colonoscopy screening uptake.    Examples of included intervention components: letter, brochure, multicomponent, navigation, phone, primary care physician counselling, video, email, and financial incentive. | Patient | Results showed that behavioural interventions increased colonoscopy completion in comparison to controls. |

*CRC= colorectal cancer, US=United States, GP=general practitioner, FOBT=guaiac faecal occult blood test, iFOBT=immunochemical faecal occult blood test, gFOBT=guaiac faecal occult blood test,*

**S7. Characteristics of included randomised controlled trials**

| **#** | **Author(s)** | **Type of RCT** | **Intervention** | **Intervention target population** | **Key outcomes** |
| --- | --- | --- | --- | --- | --- |
| 1 | Aubin-Auger et al. (2016) (16) | Cluster-randomised controlled 2-arm parallel design | The implementation of a 4-hr GP training course on communication skills and patient care to improve patient adherence and CRC participation rate. | Provider | The authors identified increased patient participation rate with iFOBT screening. |
| 2 | Cross et al. (2021) (17) | Cluster-randomised controlled 2-arm parallel design | A GP-endorsed invitation letter accompanied with a screening kit to increase participation in bowel cancer screening. | Patient | This study found that GP-endorsed invitation letter accompanied with a testing kit achieved a modest increase in bowel cancer screening participation, however it did not affect the socioeconomic inequalities in participation in the bowel cancer screening programme. |
| 3 | Dodd et al. (2019) (18) | Cluster-randomised controlled 2-arm parallel design | A multicomponent intervention that includes point-of-care FOBT provision, printed screening advice, and GP endorsement delivered in a general practice setting to increase CRC screening rates. | Patient and provider | This study found that a multicomponent intervention delivered in a general practice setting increased screening for patients overdue for screening. |
| 4 | Emery et al. (2023) (19) | Two-arm stratified randomised controlled trial | An intervention in the form of a computerised risk assessment decision support tool (CRISP) to increase risk appropriate screening, completed by GP | Patient and provider | The CRISP tool intervention identified an increase in risk-appropriate CRC screening for patients who are due for screening. |
| 5 | Guiriguet et al. (2016) (20) | Cluster randomised controlled 2-arm parallel design | An electronic reminder that alerts primary care professionals of patients who have been invited to the screening program but have not screened. | Provider | The authors found that the electronic reminder did not achieve statistical significance using ITT analysis but using a PP analysis the results were statistically significant. |
| 6 | Hirst et al. (2017) (21) | Randomised controlled 2-arm parallel design | A GP-endorsed text message reminder supporting bowel cancer screening. | Patient | This study showed that a GP-endorsed text message was effective in increasing participation for first-time screeners. |
| 7 | Irazola et al. (2023) (22) | Two-arm pragmatic cluster-randomised controlled trial | A quality improvement intervention using a PDSA model to increase CRC screening in a primary care setting. | Provider | This study found that a quality improvement-based strategy intervention was effective in increasing CRC screening. |
| 8 | Kiran et al. (2018) (23) | Randomised controlled 2-arm parallel design | A GP-endorsed reminder letter or phone call to increase uptake in CRC screening. | Patient | This study found that a GP-endorsed phone call was effective at increasing FOBT uptake in comparison to a GP-endorsed mailed reminder letter using PP analysis, as well as using ITT analysis for women. The intervention did not achieve statistical significance for men using the ITT analysis. |
| 9 | Le Breton et al. (2016) (24) | Cluster-randomised controlled 2-arm parallel design | A printed computer-generated reminder list for GPs of patients who have not scheduled an FOBT screening designed to increase patient adherence. | Provider | A computer-generated patient reminder list for GPs showed an increased trend in iFOBT uptake however the results were not statistically significant. |
| 10 | Raine et al. (2016) (25) | Cluster-randomised controlled 2-arm parallel design | A GP-endorsement letter of bowel cancer screening to improve the socioeconomic inequalities in CRC screening uptake. | Patient | The authors found that a GP-endorsed invitation letter was effective in increasing participation in CRC screening. |
| 11 | Rat et al. (2017) (26) | Cluster-randomised controlled 3-arm parallel design | Reminder list for GPs of average-risk patients of CRC who are yet to undergo screening. | Provider | This study showed that a patient-specific reminder led to a small significant increase in CRC screening participation |
| 12 | Trevena et al. (2022) (27) | Cluster-randomised controlled 2-arm parallel design | Decision support tool for patients, designed to identify barriers to risk appropriate screening for patients and CRC screening uptake. | Patient | The authors found the decision support tool was effective at increasing participation in FOBT screening. |

*GP=general practitioner, CRC=colorectal cancer, FOBT=fecal occult blood test, iFOBT=immunochemical fecal occult blood test, CRISP=Colorectal RISk Prediction, PDSA=plan-do-study-act, ITT=intention-to-treat, PP=per-protocol*

**S8. Coding of barriers to the Theoretical Domains Framework**

| **TDF domain** | **Barrier description** | **# Studies** | **References** |
| --- | --- | --- | --- |
| Knowledge | Patient lack of awareness of their risk of CRC | 1 | (27) |
|  | GP lack of awareness of recommendations for CRC screening | 1 | (27) |
| Environmental context & resources | No formal GP involvement in the population-based screening program | 3 | (22,23,25) |
|  | Lack of access to FOBT kit | 3 | (18,19,22) |
|  | Limited consultation time for providing CRC screening advice | 4 | (16,18,24,26) |
|  | Underutilisation of pop-up EMR reminders | 1 | (20) |
|  | Performance issues with pop-up EMR reminders | 1 | (18) |
|  | Sub-optimal family history records in general practice | 1 | (27) |
|  | Lack of good data and resources for generating list of nonrespondents | 1 | (26) |
| Skills | Insufficient training of GPs | 1 | (24) |
|  | Communication style not patient-centred | 2 | (16,24) |
| Memory, attention & decision processes | Patient forgetfulness | 1 | (21) |
|  | Provider forgetfulness | 1 | (24) |
| *CRC=colorectal cancer, EMR=electronic medical record, FOBT=faecal occult blood test, GP=general practitioner* | | | |

**S9. Coding of components to the Behaviour Change Technique taxonomy**

| **#** | **BCT** | **Target of BCT** | **# Studies** | **References** |
| --- | --- | --- | --- | --- |
| 1.1 | Goal setting (behaviour) | Provider | 1 | (22) |
| 1.2 | Problem solving | Provider | 2 | (19,22) |
| 1.3 | Goal setting (outcome) | Provider | 1 | (22) |
| 3.1 | Social support (unspecified) | Provider | 1 | (22) |
| 4.1 | Instruction on how to perform the behaviour | Patient, Provider | 4 | (16,18,20,26) |
| 4.2 | Information about antecedents | Provider | 1 | (19) |
| 5.1 | Information about health consequences | Patient, Provider | 4 | (18,20,23,27) |
| 6.1 | Demonstration of the behaviour | Provider | 1 | (16) |
| 6.2 | Social comparison | Provider | 1 | (16) |
| 6.3 | Information about others approval | Provider | 1 | (16) |
| 7.1 | Prompts/cues | Patient, Provider | 10 | (16–21,23,24,26,27) |
| 8.1 | Behavioural practice/rehearsal | Provider | 1 | (16) |
| 9.1 | Credible source | Patient, Provider | 8 | (17–21,23,25,27) |
| 12.1 | Restructuring the physical environment | Provider | 2 | (19,22) |
| 12.5 | Adding objects to the environment | Patient/Provider | 2 | (18,19) |

**S10. Coding of strategies to the Expert Recommendations for Implementing Change framework**

| **ERIC strategy** | **# Studies** | **References** | **Example** | **Target of strategy** |
| --- | --- | --- | --- | --- |
| Alter incentive/allowance structure | 1 | (27) | Participating GPs were eligible for CPD points for their involvement in the trial | Provider |
| Change physical structure and equipment | 1 | (18) | Provision of pre-paid iFOBT kit with return postage and pre-filled laboratory form | Patient |
| Conduct educational outreach visits | 1 | (16) | GPs received 4-hr educational training session delivered in person focusing on communication skills | Provider |
| Develop educational materials | 6 | (16,18,19,22,23,27) | The research team provided patients with a single-page CRC screening leaflet, printed in colour to Grade 8 Flesh-Kincaid, which they could show their GP during their appointment | Patient |
|  |  |  | The research team developed the ‘Which test is best’ website to provide information about CRC risk and a CRC risk assessment for patients | Patient |
| Develop and implement tools for quality monitoring | 2 | (19,22) | The CRISP tool was implemented to test the effect of using a risk assessment and decision support tool on risk-appropriate CRC screening in Australian general practice | Provider |
|  |  |  | An intervention-based improvement model in the form of a PDSA was implemented in 2-month cycles, considering barriers and facilitators to support teams to articulate change | Provider |
| Develop and organise quality monitoring systems | 2 | (19,22) | The CRISP tool is a web-based application that calculates an individual's 5-year and lifetime risk of developing CRC and recommends CRC screening | Provider |
| Distribute educational materials | 8 | (16–18,20,21,23,25,27) | Patients were provided with the single-page CRC screening leaflet when they arrived at the clinic | Patient |
|  |  |  | Patients could access information about CRC and other resources, a summary of NHMRC guidelines and an explanation of CRC risk categories | Patient |
| Engage or include patients/consumers and families in the implementation effort | 2 | (16,19) | Participants due for an iFOBT test were shown how to complete the test | Patient |
| Increase demand | 5 | (17,18,21,23,25) | GPs endorsed to patients (face-to face) during their appointment the importance of iFOBT screening | Patient |
|  |  |  | Patients received a GP-endorsed text message reminder | Patient |
|  |  |  | Patients received a GP-endorsed advance notification invitation letter in the mail | Patient |
|  |  |  | Patients received a GP-endorsed invitation letter with their iFOBT kit in the mail | Patient |
|  |  |  | Patients received a GP-endorsed phone call made by a trained undergraduate student or clerical staff | Patient |
| Identify and prepare champions | 4 | (16,20,24,26) | GPs were provided with training to help them endorse the importance of iFOBT screening | Provider |
| Intervene with patients/consumers to enhance uptake and adherence | 1 | (19) | Participants due for an iFOBT screening received an SMS reminder at 1-month to complete the test | Patient |
| Obtain and use patient/consumer and family feedback | 1 | (27) | Patients were involved in user testing the CRC risk assessment tool | Patient |
| Practice facilitation | 3 | (19,22,27) | The recruiting researchers worked with practice staff to generate lists of eligible patients | Provider |
| Prepare patients/consumers to be active participants | 3 | (18,23,27) | Patients were encouraged to take the leaflet with them into their GP and ask questions | Patient |
|  |  |  | Patients were encouraged to use the CRC risk assessment tool at home, and discuss with their GP if they had any questions | Patient |
| Remind clinicians | 6 | (16,19,20,24,26,27) | GPs received a memo summarising communication skills that had been covered in their training session | Provider |
|  |  |  | GPs received a printed list of their patients who had not undergone CRC screening | Provider |
|  |  |  | GPs received a list describing region-specific CRC screening adherence trends | Provider |
|  |  |  | GPs received an EMR alert during their patient consultation if the patient was not up to date | Provider |
|  |  |  | GPs received a copy of their patients’ CRC risk assessment report via automatic forward by fax or email | Provider |
| Unclassified | 1 | (23) | Patients had to be verified by their physician as appropriate for screening | Provider |
| *CRC=colorectal cancer, iFOBT= immunochemical faecal occult blood test, GP=general practitioner, NHMRC=National Health and Medical Research Council, CPD=continuing professional development, EMR=electronic medical records, CRISP=Colorectal RISk Prediction, PDSA=plan-do-study-act, SMS=short message service* | | | | |

**References**

1. Bellhouse S, McWilliams L, Firth J, Yorke J, French DP. Are community-based health worker interventions an effective approach for early diagnosis of cancer? A systematic review and meta-analysis. Psycho-Oncol. 2018 Apr 1;27(4):1089–99.

2. Dougherty MK, Brenner AT, Crockett SD, Gupta S, Wheeler SB, Coker-Schwimmer M, et al. Evaluation of Interventions Intended to Increase Colorectal Cancer Screening Rates in the United States: A Systematic Review and Meta-analysis. JAMA Int Med. 2018 Dec 1;178(12):1645–58.

3. Facciorusso A, Demb J, Mohan BP, Gupta S, Singh S. Addition of Financial Incentives to Mailed Outreach for Promoting Colorectal Cancer Screening: A Systematic Review and Meta-analysis. JAMA Netw Open. 2021 Aug 25;4(8):e2122581–e2122581.

4. Goodwin BC, Ireland MJ, March S, Myers L, Crawford-Williams F, Chambers SK, et al. Strategies for increasing participation in mail-out colorectal cancer screening programs: a systematic review and meta-analysis. Systematic Reviews. 2019 Nov 4;8(1):257.

5. Jager M, Demb J, Asghar A, Selby K, Mello EM, Heskett KM, et al. Mailed Outreach Is Superior to Usual Care Alone for Colorectal Cancer Screening in the USA: A Systematic Review and Meta-analysis. Dig Dis Sci. 2019 Sep 1;64(9):2489–96.

6. Lau J, Ng A, Wong GJ, Siew KY, Tan JKH, Pang Y, et al. How effective are digital technology-based interventions at promoting colorectal cancer screening uptake in average-risk populations? A systematic review and meta-analysis of randomized controlled trials. Prev Med. 2022 Nov;164:107343.

7. Long NN, Lau MPXL, Lee ARYB, Yam NE, Koh NYK, Ho CSH. Motivational Interviewing to Improve the Uptake of Colorectal Cancer Screening: A Systematic Review and Meta-Analysis. Front Med. 2022;9.

8. Myers L, Goodwin B, March S, Dunn J. Ways to use interventions to increase participation in mail-out bowel cancer screening: a systematic review and meta-analysis. Transl Behav Med. 2020 May 20;10(2):384–93.

9. Posadzki P, Mastellos N, Ryan R, Gunn L, Felix L, Pappas Y, et al. Automated telephone communication systems for preventive healthcare and management of long‐term conditions. Cochrane Database Syst Rev. 2016;(12).

10. Ramli NS, Manaf MR, Hassan MR, Ismail MI, Nawi AM. Effectiveness of Colorectal Cancer Screening Promotion Using E-Media Decision Aids: A Systematic Review and Meta-Analysis. Int J Environ Res Public Health. 2021;18(15).

11. Rana T, Chan D, Nguyen K, Choi KC, So WKW. Effectiveness of community health worker–led interventions in improving the CRC screening uptake rate in racial and ethnic minority populations. Cancer Nursing. 2023;

12. Rubin L, Okitondo C, Haines L, Ebell M. Interventions to increase colorectal cancer screening adherence in low-income settings within the United States: A systematic review and meta-analysis. Preventive Medicine. 2023;172.

13. Tsipa A, O’Connor DB, Branley-Bell D, Day F, Hall LH, Sykes-Muskett B, et al. Promoting colorectal cancer screening: a systematic review and meta-analysis of randomised controlled trials of interventions to increase uptake. Health Psychology Review. 2021 Jul 3;15(3):371–94.

14. Volk RJ, Linder SK, Lopez-Olivo MA, Kamath GR, Reuland DS, Saraykar SS, et al. Patient Decision Aids for Colorectal Cancer Screening: A Systematic Review and Meta-analysis. Am J Prev Med. 2016 Nov 1;51(5):779–91.

15. Yakoubovitch S, Zaki T, Anand S, Pecoriello J, Liang PS. Effect of Behavioral Interventions on the Uptake of Colonoscopy for Colorectal Cancer Screening: A Systematic Review and Meta-Analysis. Am J Gastroenterol. 2023 Oct 1;118(10):1829–40.

16. Aubin-Auger I, Laouénan C, Le Bel J, Mercier A, Baruch D, Lebeau JP, et al. Efficacy of communication skills training on colorectal cancer screening by GPs: a cluster randomised controlled trial. Eur J Cancer Care. 2016 Jan;25(1):18–26.

17. Cross AJ, Myles J, Greliak P, Hackshaw A, Halloran S, Benton SC, et al. Including a general practice endorsement letter with the testing kit in the Bowel Cancer Screening Programme: Results of a cluster randomised trial. J Med Screen. 2021 Dec;28(4):419–25.

18. Dodd N, Carey M, Mansfield E, Oldmeadow C, Evans TJ. Testing the effectiveness of a general practice intervention to improve uptake of colorectal cancer screening: a randomised controlled trial. Australian and New Zealand Journal of Public Health. 2019;43(5):464–9.

19. Emery JD, Jenkins MA, Saya S, Chondros P, Oberoi J, Milton S, et al. The Colorectal cancer RISk Prediction (CRISP) trial: a randomised controlled trial of a decision support tool for risk-stratified colorectal cancer screening. Br J Gen Pract. 2023 Aug;73(733):e556–65.

20. Guiriguet C, Muñoz-Ortiz L, Burón A, Rivero I, Grau J, Vela-Vallespín C, et al. Alerts in electronic medical records to promote a colorectal cancer screening programme: a cluster randomised controlled trial in primary care. Br J Gen Pract. 2016 Jul;66(648):e483-490.

21. Hirst Y, Skrobanski H, Kerrison RS, Kobayashi LC, Counsell N, Djedovic N, et al. Text-message Reminders in Colorectal Cancer Screening (TRICCS): a randomised controlled trial. British Journal of Cancer. 2017 May 1;116(11):1408–14.

22. Irazola V, Santero M, Sanchez M, Tristao I, Ruiz JI, Spira C, et al. Quality improvement intervention to increase colorectal cancer screening at the primary care setting: a cluster-randomised controlled trial. BMJ Open Qual. 2023 Jun;12(2):e002158.

23. Kiran T, Davie S, Moineddin R, Lofters A. Mailed Letter Versus Phone Call to Increase Uptake of Cancer Screening: A Pragmatic, Randomized Trial. J Am Board Fam Med. 2018 Dec;31(6):857–68.

24. Le Breton J, Ferrat É, Attali C, Bercier S, Le Corvoisier P, Brixi Z, et al. Effect of reminders mailed to general practitioners on colorectal cancer screening adherence: a cluster-randomized trial. Eur J Cancer Prev. 2016 Sep;25(5):380–7.

25. Raine R, Duffy SW, Wardle J, Solmi F, Morris S, Howe R, et al. Impact of general practice endorsement on the social gradient in uptake in bowel cancer screening. Br J Cancer. 2016 Feb 2;114(3):321–6.

26. Rat C, Pogu C, Le Donné D, Latour C, Bianco G, Nanin F, et al. Effect of Physician Notification Regarding Nonadherence to Colorectal Cancer Screening on Patient Participation in Fecal Immunochemical Test Cancer Screening: A Randomized Clinical Trial. JAMA. 2017 Sep 5;318(9):816–24.

27. Trevena L.J., Meiser B., Mills L., Dobbins T., Mazza D., Emery J.D., et al. Which Test Is Best? A Cluster-Randomized Controlled Trial of a Risk Calculator and Recommendations on Colorectal Cancer Screening Behaviour in General Practice. Public Health Genomics. 2022;((Trevena, Naicker) Faculty of Medicine and Health, School of Public Health, University of Sydney, Sydney, NSW, Australia):1–16.
